# American Dentistry Abroad

**Published:** 1898-08

**Authors:** L. C. Bryan

**Affiliations:** Basel and Davos, Switzerland


### AMERICAN DENTISTRY ABROAD.

BY L. C. BRYAN, D.D.S., F.B.D.C., ETC., BASEL AND DAVOS, SWITZERLAND.

Having figured for some years on the staff of your journal as foreign correspondent, I take the liberty of exercising my function in calling your attention, and, through your widely circulated columns, the attention of the dental public at large, to a matter of universal importance to all American dentists at home and abroad. We have watched with growing interest as the years have passed the important improvements in the status of American dental institutions of learning, and especially of the ever-growing importance of the National Association of Dental Faculties, and the good work it is accomplishing in an ever-increasing sphere of importance and usefulness and ever-rising standard of requirements.

As usual, America is still in the van in this as in all dental matters, and I do not know that any such powerful organization in the sphere of education exists elsewhere in the world, and certainly nowhere with such a wonderful future for usefulness before it. It is an organization unique and wonderful in its conception and development, and its importance as a means of regulating and controlling the elaborate system of American dental education is perhaps not generally understood. In a few years this organization has taken unto itself one by one every college of any note in America, so that now if there is any question of the standing of any dental college the only thing necessary to do is to turn to the list of colleges in the National Association of Dental Faculties, and if its name is there no further questions are asked. "Race, color, or previous condition of servitude" (to any former clique controlled by money-making managers) has now no bearing on the standing of a member of the Association. Its standard is known, published to the world, and its members must live up thereto. A new era has opened for American dentistry. The great trusts set the example of what could be done by concerted action and co-operation, and the dental colleges have taken all the good there is in a trust organization, and dentistry does not yet know what that may bring forth for good. Colleges that a few years ago were open to any plausible business proposition from a young man with cash are now enrolled under its rules and regulations, and the young man with cash and his business proposition is simply shown a list of requirements of the National Association of Dental Faculties, and has to accept the terms it proposes, and has no other alternative. This is remarkable, considering that a few years ago he could, by judicious "shopping" and with a proper tip from a friend or two, have made his own terms with some very respectable and old institutions of dental learning. Now, if this young man without any qualifications but cash arrives on a European steamer too late for matriculation and applies for admission to the "oldest" dental college and is refused matriculation, and then succeeds by "gross misrepresentation" (?) in getting matriculated across the way in a highly respectable university, and writes home to his friends that he is in the senior class of that highly respectable university, with two hundred and fifty dental students, and will be back in June with an American diploma, it is only necessary for those interested in the good name of American dentistry to protest and give the facts to this great National Association, and the young man is within a few days reduced to the rank of freshman, and writes home that he will not return to his friends for three years. And this year the highly respectable university may be called on to explain to the Association how the young man could be matriculated at all after arriving by the European steamer on the afternoon of October 24 and having been refused matriculation in the oldest on October 28, and if it cannot explain how this happened there will be trouble ahead for the highly respectable university.

At the last meeting of the National Association of Dental Faculties a committee was appointed to investigate the requirements of foreign dental institutions, with a view of getting recognition of the American degrees by foreign countries and *vice versa*, and of acquiring other information, and this it is doing in an energetic manner through Dr. Barrett as chairman. The work of this committee, if anything is accomplished, will be the most important in the history of the Association. I have suggested (see *Dental Review*, February 15, 1898) that one of its first functions should be the selection of preliminary examiners, one or more in each foreign country, as may be decided, and each applicant for admission to American dental colleges shall bring a certificate from the examiner in his country as to the value of the certificates he presented, as judged by American standards, which will be set by the Association. Should the candidate be or have arrived in America, his certificates shall be submitted to the examiner of his country before matriculating him. These examiners will inform candidates into what class of any American college in affiliation with the National Association of Dental Faculties they may enter, and shall give them letters to this effect, and their decision as to the value of these certificates shall be final. This would prevent "shopping" to secure best rates, and would also prevent much of the easy graduation of foreigners, which has been such a disgrace to the American diploma abroad. The foreigner, in many cases, seeks the school in which he can graduate in one term, and his main object is to return to his own country as soon as possible and practise under the name of American dentist. He does not go to learn all there is to be learned of American dentistry, but merely to get the title as expeditiously as may be, and few of such adventurers have required more than a few months to return a D.D.S., whereas a native American never gets through with less than the whole prescribed course of the college. This is mainly owing to the presentation of certificates in a foreign language by applicants from other countries, with seals and other paraphernalia, which the deans of the colleges have not taken the trouble to investigate or have not comprehended the full value or meaning of. Irregular graduations have of later years become more difficult, but still a candidate seldom fails to find an institution of some kind that will accept his terms, and it is all the same to him whether it is a recognized college, connected with the National Association of Dental Faculties, or a private institution, a free lance, in fact, such as Chicago can now show, which rivals in infamy Buchanan's diploma-mill of accursed memory. I have in my hands now, received to-day from a Swiss college, a circular of one of these Illinois mills.

The great difficulty about the recognition of American degrees by foreign authorities has been the inability to distinguish between high and low standard colleges, and it should be made plain to them that no college is recognized in America which does not comply with the standard of requirements of the National Association of Dental Faculties. Theirs should be the universal standard.

The State Department at Washington should be shown the importance and the influence and position of American dentistry in foreign countries, securing as it does a representative resident in the country, acquainted with its institutions, influential for good among its people, an educated professional man, ready to work for American interests and not changing with every administration, having generally an income sufficient to enable him to invest in his country's industries and enterprises and in this way support them, and finally support himself in comfort in his native land. Senator Hale once said that in all his travels abroad he had found the resident American dentists the best representatives of their country whom he had met.

The American ministers abroad should be instructed as to the values of diplomas of the different institutions, given a list of those which the State boards recognize, or of those which live up to the highest standard, and more important still, be given a list of those which are *not* recognized colleges. These should be communicated to the various governments and to the authorities of the foreign dental colleges, which are just now beginning the work long done by old established American institutions.

The State Department has recently collected, through its foreign representatives, the full text of the laws of all foreign countries, and this is accessible to any one who will write for it to the Department, and is a good basis to work on.

The present standard of requirements of our schools is such, except in preliminary education, that no foreign country can object to receiving our graduates on an equal footing with their own and giving them equal privileges, which few, if any, in Europe now do, so far as I know. When the preliminary studies standard is raised there will be no possibility of refusing our graduates.

The laws as laid down in most countries are almost exclusive; but if a man has the means to live and the will to study he may overcome all difficulties, and good operators will always find a good opening, if not as an established dentist with full rights, at least as assistant.

The requirements in several countries, as will be seen in the March number of the INTERNATIONAL DENTAL JOURNAL, is the possession of a medical diploma. This means three to seven years study in the country and a good previous education. However, in countries like Austria, a good American dentist can associate himself with a medical man of the country and practise dentistry with great profit and success. In these countries, if a medical man is unsuccessful as a physician, or has a taste for dentistry, it is a common custom for him to set up as a dentist without further study of the diseases of the mouth and teeth than he can get out of such text-books on this specialty as his country affords. What a blessing for his patients could he secure as assistant the services of a thoroughly trained American dentist! And this they often do if one is available. Salaries in Europe are from fifteen hundred to three thousand dollars, and some assistants make from four thousand to five thousand dollars on shares, when capable.

Any one can establish himself in Germany and put out his plate as Amerikan dentist (with a k), but must not call himself doctor or Zahnarzt. The Zahnarzt is one who has passed his examinations in Germany and has rights as a doctor. The right to labor is free to all (*Gewerbe Freiheit*).

Switzerland requires a preliminary education called the "maturity," "made in Switzerland," which is about equal to our B.A. degree, and necessitates a residence in the country of from one to four years, long enough to learn either the French or German language, and then to make the studies prescribed for the maturity. After this is met the dental examination consists in passing in certain medical and other scientific branches, and putting in three fillings, one of which must be gold, and making a plate. Former home studies are not given credit for, and *all* must be made in Switzerland.

The Medical Council or Leitende Ausschuss have had the option of giving the maturity, but this favor is only granted to Swiss, and *all* must be able to make a simple gold filling, etc., and pass the practical examinations in full.

One advantage foreign schools have over American ones is that if they do not all teach *dentistry* as fully and successfully as American colleges, they require a higher standard of preliminary education. Ours have looked more to the making of practical dentists than to the turning out of highly educated professional men, and in this respect we could learn something from the foreign colleges.

As long as American colleges recognize the degrees of foreign dental colleges and allow applicants from them to enter the senior class while possessing only foreign diplomas, there will be no chance for the recognition of American degrees in the countries where they are now not accepted, and where the American dentist, with his D.D.S. has to make the whole studies from the beginning, with the difficulties of language in his way; and we can get no reciprocity as long as this condition of things exists. American graduates must make the full course in any of the institutions here I know of, and must make their preliminary examination, "the maturity," and something ought to be done so that American graduates, fully equipped for their profession, shall be admitted on more favorable terms to a course in a foreign college, or else some course of study should be prescribed to the foreign student in America equal to that imposed on Americans abroad generally, if we cannot raise our own preliminary standard higher just yet. But why not?

Therefore I say that the work of the National Association of Dental Faculties committee is very important and has a great task ahead of it, which, let us hope, it may accomplish to the end that Americans may find a foothold in foreign countries where recent laws have made it almost impossible for them to enter, and agitations are going on to increase the difficulties even more for the "foreigner" from America.

